# Cortical Representation of Tactile Stickiness Evoked by Skin Contact and Glove Contact

**DOI:** 10.3389/fnint.2020.00019

**Published:** 2020-04-09

**Authors:** Junsuk Kim, Isabelle Bülthoff, Heinrich H. Bülthoff

**Affiliations:** ^1^Department of Human Perception, Cognition and Action, Max Planck Institute for Biological Cybernetics, Tübingen, Germany; ^2^Department of Industrial ICT Engineering, Dong-Eui University, Busan, South Korea

**Keywords:** direct touch, indirect touch, tactile stickiness, tactile intensity encoding, fMRI

## Abstract

Even when we are wearing gloves, we can easily detect whether a surface that we are touching is sticky or not. However, we know little about the similarities between brain activations elicited by this glove contact and by direct contact with our bare skin. In this functional magnetic resonance imaging (fMRI) study, we investigated which brain regions represent stickiness intensity information obtained in both touch conditions, i.e., skin contact and glove contact. First, we searched for neural representations mediating stickiness for each touch condition separately and found regions responding to both mainly in the supramarginal gyrus and the secondary somatosensory cortex. Second, we explored whether surface stickiness is encoded in common neural patterns irrespective of how participants touched the sticky stimuli. Using a cross-condition decoding method, we tested whether the stickiness intensities could be decoded from fMRI signals evoked by skin contact using a classifier trained on the responses elicited by glove contact, and vice versa. Our results found shared neural encoding patterns in the bilateral angular gyri and the inferior frontal gyrus (IFG) and suggest that these areas represent stickiness intensity information regardless of how participants touched the sticky stimuli. Interestingly, we observed that neural encoding patterns of these areas were reflected in participants’ intensity ratings. This study revealed common and distinct brain activation patterns of tactile stickiness using two different touch conditions, which may broaden the understanding of neural mechanisms related to surface texture perception.

## Introduction

We can perceive surface texture in various ways. To perceive the surface properties of sandpaper, we can touch and explore its surface with our bare skin. In this direct touch, the fingertip directly contacts the rough surface and the roughness information is delivered from the skin deformation that is indented from its resting position (Taylor and Lederman, 1975). Also, we can recognize the surface roughness by moving a rigid stick over the sandpaper (Klatzky and Lederman, 1999). In this indirect touch, roughness information is perceived by the cues transmitted along with the physical link (the rod) between textured surfaces and skin. Although these two kinds of tactile exploration are physically very different, both enable us to perceive surface characteristics (Klatzky and Lederman, 1999; LaMotte, 2000; Klatzky et al., 2003). The indirect touch can also be observed in the domain of tactile stickiness perception. For instance, when we touch a sticky surface (e.g., the back of a post-it note) with the skin of fingertip, we perceive a sticky sensation when the skin begins detaching from the surface (Zigler, 1923). We can also perceive a surface as sticky when we touch it while wearing a glove. In this case, when the finger is lifted off, there are several influences on the stickiness perception, for example, a pressure stimulus applied on the back of the finger through the glove, a change in the contact between the glove and the finger skin, and neural signals from the muscle and joint afferents. Similar to the aforementioned roughness perception using a rod, these two kinds of stickiness explorations deliver physically different stimulations in terms of stimulated location (fingertip vs. back of the finger) and stimulus action onto the fingertip (skin stretching vs. skin depression). Nonetheless, we are still able to perceive that the surface is sticky, despite some perceptual distortion or intensity weakening.

To broaden our understanding regarding the aspects of touch, several psychophysical and neurophysiological studies have investigated surface texture encoding and perception (Connor et al., 1990; Phillips et al., 1992; Blake et al., 1997). These studies have shown that touch is highly complex and includes a wide range of perceptions (Hollins et al., 1993; Hollins and Roy, 1996) and that the neural responses from different types of afferents enable us to perceive a wide range of surfaces (Mackevicius et al., 2012; Weber et al., 2013). Among the various aspects of tactile perception of texture surfaces (Okamoto et al., 2013), relatively little is known about stickiness and its neural mechanism (Bensmaia, 2016). Based on the previous findings (Olausson et al., 2000; Bensmaia, 2016), it is likely that both the slowly adapting afferents type 1 and 2 (SA1 and SA2) would contribute to stickiness perception and all mechanoreceptive afferents respond to some degree in assessing stickiness. At the cortical level, several neuroimaging studies have investigated the neural encodings of tactile stickiness perception when the surface is touched by a bare fingertip (Kim et al., 2017; Yeon et al., 2017). Our previous human fMRI study found that neural activity patterns in the posterior parietal cortex represent the perceptual intensities of sticky surfaces (Kim et al., 2017). Moreover, a recent psychological study investigated how cues in different sensory modalities (auditory, tactile, and visual cues) influence stickiness perception and revealed that texture information processing in the auditory domain is distinctive from that of other modalities (Lee et al., 2019). However, there is as yet no research identifying and comparing brain activities triggered by direct (i.e., skin contact) and indirect (i.e., glove contact) touch. More specifically, it remains unknown whether stickiness information evoked in both touch conditions is encoded in similar patterns in the brain.

## Materials and Methods

### Participants and Ethics Approval

Eighteen healthy volunteers (14 females, average 24.4 ± 2.8 years old) with no contraindications to MR investigations and no history of neurological disorders participated in the experiment. All participants were right-handed and had no deficits in tactile processing. Experimental procedures were approved by the ethical committee of the University Clinics Tübingen (649/2016BO2) and the study was conducted following the Declaration of Helsinki. All participants were informed about the experimental procedure and gave written informed consent before their participation.

### Tactile Stimuli

**Figure 1:**
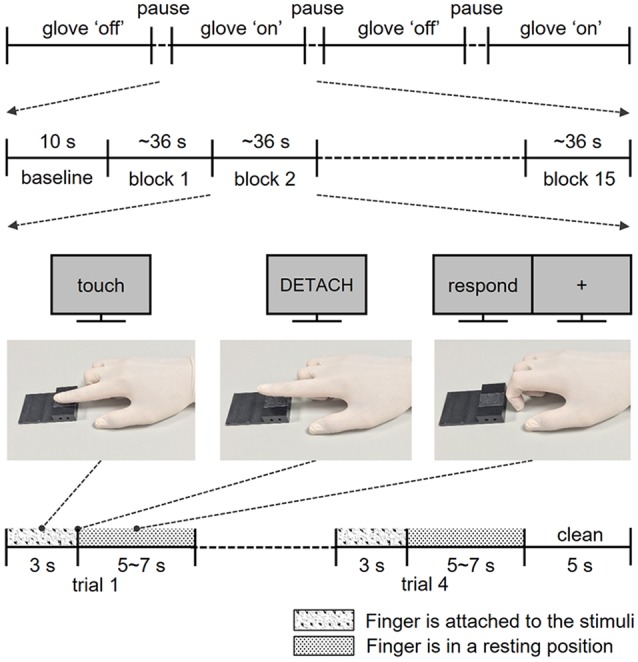
Structure of the experiments. Participants completed four fMRI runs, two runs for the skin contact condition and two runs for glove contact condition. In each run, participants performed a total of 60 trials in 15 blocks. Following the instructions on the screen, participants touched and lifted their right index finger off the sticky surface. After the exploration, participants rated the perceived stickiness intensity level from 1 to 5 with their left hand.

Three different 3 M double-sided repositionable tape (9415PC, 665, and 9425; 3 M Center, St. Paul, MN, USA) were prepared in the size 5 × 1.9 cm and attached to a plastic-plate sized 5 × 8 cm (Figure 1). The plastic plate was used to enable the experimenter to present the stimuli easily without direct contact with them. The tapes were presented with the test sticky side facing up (the glue on the other side secured the tape onto the support). All stimuli were used only once, they were replaced by a fresh one of the same stickiness level. The sticky material of those tapes was manufactured to allow easy removal of the tapes. It was harmless to the skin and did not evoke any painful sensation. Indeed, at the post-experiment interview, no participant reported that stimuli were uncomfortable.

According to the technical data sheet provided by the manufacturer, the physical stickiness intensities of the tapes 9415PC, 665, and 9,425 measured by a “180° peel-off test” were 5.4, 25, and 13 N/100 mm, respectively. This “peel-off test” is one of the methods testing adhesion properties and it measures the mean force required to remove the tape at a steady rate. As the main purpose of this test is to evaluate the uniformity of the adhesion properties of the surface, we do not think that it does relate well to how humans perceive the stickiness of those tapes during our experiment. To give an estimate of the physical stickiness of those tapes, we considered rather the “probe tack test” to approximate more properly human stickiness perception. This test focuses on the peak value of adhesive force, thus indicating instantaneous adhesion property. In previous studies (Mith et al., 2008; Cakmak et al., 2011), this test was considered as a good approach to evaluating human stickiness sensations. Using a probe in the shape of a stainless steel ball (1-inch diameter), we recorded the peak value of the adhesive force that occurs when a probe is peeled off at a peeling rate of 2 mm/s. The physical stickiness intensities of 9415PC, 665, and 9,425 were estimated as 3.3, 35.6, and 43.9 N/100 mm, respectively. Based on these measured values, we sorted the tapes in ascending order of stickiness ball intensity. Despite the nonlinear increase of the intensity, participants could distinguish each of them clearly (see the “Results” section). We added a “No-tape” condition. In this condition, participants touched a plastic plate without a tape (not sticky at all) instead of a sticky surface. Consequentially, the following four different physical stickiness intensities were used in the experiment: no-tape (level 0), 9415PC (level 1), 665 (level 2), and 9,425 (level 3).

Disposable latex gloves (with a mean thickness of 0.08 mm) were used for the glove contact condition. According to the technical data sheet provided by the manufacturer (Latex-OC55, BINGOLD GmbH + Co. KG, Hamburg, Germany), these powder-free gloves were micro-textured on finger sections and offered enhanced sensitivity on tactile perception. Four different sizes of gloves (S, M, L, and XL) were available to provide tight-fit gloves to each participant.

### Experimental Procedures

#### Training Session

Before the main experiment, participants performed three training sessions outside the MR room. First, participants practiced “Touch,” “Detach,” and “Rest” finger postures following instructions of the experimenter to standardize their finger movements to minimize confounding factors due to movement variations across participants as well as across trials (Figure 1). Second, participants were trained to exert a pressure force of 1 N consistently. The equalization of the exerted force is essential to evoke a similar level of sticky sensation because the perceived stickiness intensity of a stimulus is closely related to the force exerted perpendicular to the surface (Bensmaia, 2016). In this training session, participants pressed their right index fingertip on a pressure sensor (A201-100, FlexiForce, MA, USA). A pressure value displayed on the monitor screen gave them feedback so that they could practice applying the correct pressure. Third, to familiarize the participants with the different levels of the stickiness of the stimuli and to obtain an estimation of their perception, participants verbally reported the perceived stickiness intensity of each stimulus on a scale from 1 (not at all sticky) to 5 (most sticky) in a rating task. This task consisted of 40 trials (2 touch conditions × 4 stickiness intensities × 5 repetitions) and mimicked closely how participants touched the stimuli during the main experiment, except for participant’s position (sitting instead of laying on their back). Note that the responses in this training session were not analyzed and we never mentioned the number of intensity levels throughout the entire experiment.

#### Scanning Session

During the functional image acquisition, participants laid supine in the MR scanner with their right arm comfortably placed along the magnet bore. They wore earplugs and watched a computer screen with a projector resolution of 1,280 × 1,024 pixels at a refresh rate of 60 Hz *via* an angled surface-mirror. An experimenter was positioned at the entrance of the magnet bore and placed a new stimulus plate on the MR table under the participant’s finger for each trial. Functional MRI data were acquired in four runs: two runs for the skin contact condition (bare skin) and two runs for the glove contact condition (gloved hand; Figure 1). Each run started with a 10 s baseline period during which the finger remained in the rest position (Figure 1) and 60 trials were presented in 15 blocks of four trials (four stickiness intensities × 15 repetitions). Stimulus intensities in each block were presented in a random order. Each trial consisted of the three distinct finger postures learned in the training session: as soon as the visual instruction “Touch” appeared on the screen, participants moved their right index fingertip from the rest position to touch the stimulus with the same force as during the training and maintained that posture for 3 s. Participants detached their finger from the surface as soon as “Detach” was displayed on the screen and when “Rest” appeared, they placed their finger on the table for 5–7 s until the next trial. During the “Rest” period, participants were asked to rate the perceived stickiness intensity level from 1 to 5 and the stimulus plate was replaced by the experimenter for the next trial. To remove any sticky material from the finger, the experimenter cleansed the fingertip with a sponge imbibed with rubbing alcohol for 5 s at the end of each block. The duration of each trial was approximately 9 s and each run lasted about 10 min and 30 s.

### MR Data Acquisition and Preprocessing

Neuroimaging data were acquired on a 3 Tesla Siemens Prisma system with a 64-channel head coil (Siemens Medical Systems, Erlangen, Germany). Anatomical images were obtained using a T1-weighted sequence (ADNI, 192 slices) with the following parameters: Repetition time (TR) = 2,000 ms, echo time (TE) = 3.06 ms, flip angle = 9°, field of view (FOV) = 256 mm, and voxel size = 1 mm^3^. Functional images were acquired using a slice-accelerated multiband gradient-echo-based echo-planar imaging (EPI) sequence using T2*-weighted blood oxygenation level-dependent (BOLD) contrast (multiband acceleration factor: 2): 46 slices, TR = 1,520 ms, TE = 30 ms, flip angle = 68°, FOV = 192 mm, slice thickness = 3 mm, and in-plane resolution = 3 × 3 mm^2^. The functional images covered the whole cerebrum. Standard preprocessing of the fMRI data was performed using the Statistical Parametric Mapping package (SPM8; Wellcome Department of Imaging Neuroscience, UCL, London, UK) and a high-pass filter of 1/128 Hz was used to remove low-frequency noise. The EPI data were corrected for slice-timing differences, realigned for motion correction, co-registered to the individual T1-weighted images, normalized to the Montreal Neurological Institute (MNI) space, and spatially smoothed by a 2-mm full-width-half-maximum (FWHM) Gaussian kernel.

### fMRI Data Analysis

Two fMRI data analyses were carried out to explore various aspects of neural responses to skin contact and glove contact conditions. First, an information-based brain mapping using a cubical searchlight was employed to seek brain regions that generated distinct neural patterns in response to each stickiness intensity (Kriegeskorte et al., 2006). Specifically, the volume-based searchlight analyses were performed to discriminate three different stickiness levels (levels 1, 2, and 3) for each condition separately. Note that we excluded level 0 in the analyses to eliminate the potential influence of surface material difference. As participants were paying attention to the stickiness intensity, when stimulus level 0 was presented, they might perceive an unexpected material difference (non-sticky plastic surface vs. the surface of tape). Second, we tested whether brain regions would encode intensities regardless of touch conditions. In particular, we utilized a cross-condition classification method to look for a common spatial pattern of brain activity (Kim et al., 2019).

#### Searchlight Analyses within Each Touch Condition

We first extracted parameter estimates of the voxel response to each stickiness intensity using a GLM. The moments of “Detach” were considered as events and convolved with the canonical hemodynamic response function (HRF) of SPM8. To increase the number of examples, each regressor modeled an individual trial and a total of 120 event-related regressors (4 stimulus intensities × 15 repetitions × 2 fMRI runs) were used to predict the voxel response for each condition. Six nuisance regressors for head movement correction (movement and rotation along the three orthogonal axes) and physiological noise regressors were also defined. Obtained trial-specific parameter estimates were normalized to achieve centering relative to the mean and unit variance and then used as input features for a multivoxel pattern analysis (MVPA) implemented in the SearchMight Toolbox (Pereira and Botvinick, 2011).

We selected a searchlight of a 5 × 5 × 5 voxel cube that runs through the whole brain. In each searchlight, a Gaussian Naïve Bayes (GNB) classifier decoded the three intensity levels (levels 1, 2, and 3) from the neural activity patterns using a cross-validation procedure. We performed two searchlight analyses: (a) analysis of data obtained when touching the tape with a bare fingertip; and (b) analysis of data obtained when touching with a gloved hand. Each experimental run was considered as one-fold and a two-fold cross-validation procedure was employed. The resulting classification performances from both folds were averaged and assigned to the center voxel of the searchlight. Chance-level accuracy (0.33 in this case) was subtracted from the value stored in each voxel to yield deviations from chance. These individual accuracy maps were submitted to the random-effects group analysis. To determine significant clusters corrected for multiple comparisons, we estimated an empirical cluster size threshold in searchlight accuracy maps for a sub-group of participants chosen randomly. Following the sign-flipping permutation procedure (Oosterhof et al., 2010), we compared the size of the clusters obtained from the analysis to a reference distribution of clusters that one would obtain by chance. If stickiness intensity does not affect, searchlight accuracy values would be distributed between −0.33 and 0.67 after chance level subtraction. Namely, under the null hypothesis, the sign of the searchlight accuracies would be “+” or “−” with a probability of 67% and 33%, respectively. To estimate how large clusters could be formed under this condition, we randomly flipped the sign of the searchlight accuracy maps of a random number of participants. These maps were considered as samples under the null hypothesis and a random-effect analysis on these maps calculated the largest size of the cluster. The distribution of the largest cluster sizes under the null hypothesis was obtained from 1,000 repetitions. In this study, we considered as significant clusters if their size was within the top 5% of the upper tail of the null distribution.

#### Searchlight Analyses Between Touch Conditions

In the searchlight analyses within each touch condition, we attempted to find brain regions in which local activation patterns allowed the successful decoding of the three stickiness intensities for each condition separately. In contrast, in this searchlight analysis, we explored whether there are brain regions that represent the stickiness intensities irrespective of the specific cues conveying the sticky sensation. To identify such potential brain regions, a cross-condition classification method was utilized. All analysis steps were identical with those of the searchlight analysis within each touch condition except for the cross-validation procedure. The cross-condition classifiers were trained on all trials of the one-touch condition and tested on all trials of the other touch condition, and vice versa. The decoding performances from both cross-validation steps were averaged together. If this analysis identified brain regions displaying significant decoding performances, it means that these regions encode stickiness intensity information in similar spatial patterns for both skin contact and glove contact conditions. Moreover, to test whether the identified brain regions decoded stickiness intensities with a similar level of performance for each fold, we computed the confusion matrices for each cross-validation step.

## Results

**Figure 2:**
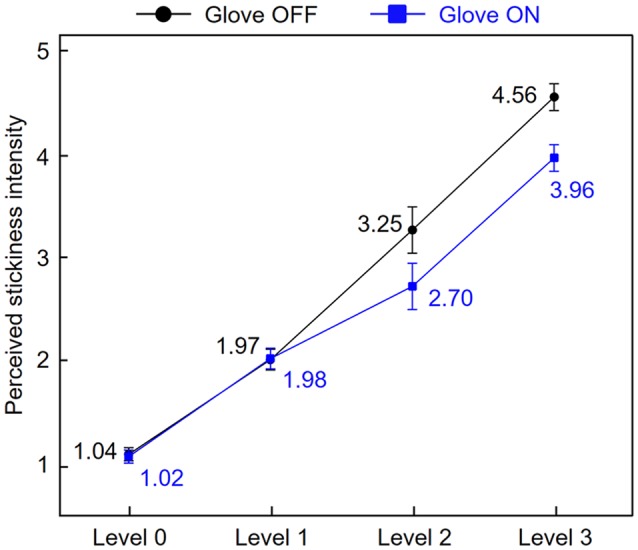
Stickiness intensity perception by skin contact and glove contact. Subjective ratings of perceived stickiness intensity for each level were averaged across participants. Error bars represent standard errors.

Before the fMRI data analysis, to investigate how similarly stickiness intensities were perceived by skin contact and glove contact, we averaged subjective ratings of perceived stickiness intensity for each level across participants (Figure 2). A two-way repeated-measures ANOVA was conducted to assess the effect of the touch conditions on the participants’ ratings across the intensity levels. There were main effects of both touch condition (*F*_(1,17)_ = 2.15, *p* < 0.01) and intensity level (*F*_(3,51)_ = 167.35, *p* < 0.01) and the interaction was also significant (*F*_(3,51)_ = 5.56, *p* < 0.01). Tukey’s *post hoc* test showed that when participants touched the sticky surface of levels 0 and 1, there was no significant difference in the rating scores between conditions (all *p* > 0.17). When participants touched the sticky surface of levels 2 and 3, there were significant differences in the rating scores between conditions (all *p* < 0.03; Tukey’s test). Also, participants perceived the four stickiness levels as distinct from each other for both touch conditions (all *p* < 0.01; Tukey’s test). These results indicate that stickiness levels 0 and 1 were perceived similarly regardless of conditions, while levels 2 and 3 were perceived distinctly between conditions. Specifically, levels 2 and 3 were perceived as less sticky when participants touched the tapes with a gloved hand instead of their bare fingertip.

### Searchlight Analyses Within Each Touch Condition

This searchlight analyses aimed to identify brain activation patterns encoding stickiness intensity information and to determine the common or distinct regions between touch conditions, for example, skin contact and glove contact. Note that it is very unlikely that any voxel cluster identified with our searchlight analyses presented below would occur by chance. A permutation procedure (Oosterhof et al., 2010) revealed that the probabilities of obtaining clusters as large as ours by chance were less than 5%.

**Figure 3:**
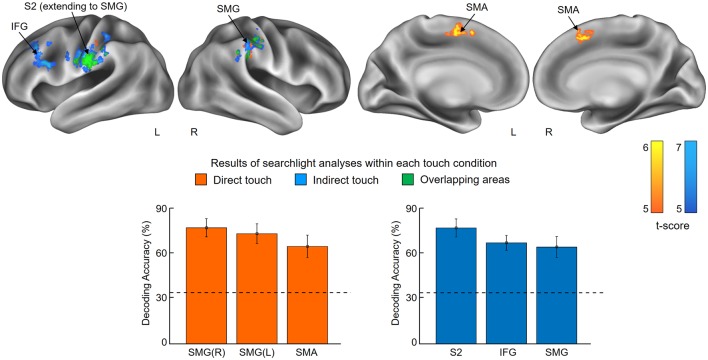
Results of searchlight analyses within each touch condition. The searchlight multivoxel pattern analysis (MVPAs) identified voxel clusters showing significant decoding performances for the stickiness intensity classification for each touch condition, i.e., skin contact and glove contact. The clusters identified from skin contact and glove contact are colored in orange and blue, respectively. Overlapping areas are highlighted in green. The lower part of the figure shows the classification performances of each identified cluster. Chance level is indicated by the dashed line (33%) and error bars indicate standard errors. Abbreviations: SMG, supramarginal gyrus; SMA, supplementary motor area; S2, secondary somatosensory cortex; IFG, inferior frontal gyrus.

The first searchlight analysis on our data obtained in the skin contact condition found three significant clusters (*p* < 0.001 uncorrected, size >100). Two are located in the SMG in the bilateral hemisphere and the third one is found in the supplementary motor area (SMA; Figure 3 and Table 1). Decoding accuracies obtained from each identified cluster were as follows (presented as mean ± standard deviation): 74.5 ± 5.5% for the ipsilateral SMG, 70.3 ± 5.9% for the contralateral SMG, and 61.6 ± 6.9% for the SMA. The second searchlight analysis in the glove contact condition revealed three significant clusters (*p* < 0.001 uncorrected, size >100) located in the secondary somatosensory cortex (S2) extending up to the SMG in the contralateral hemisphere, the inferior frontal gyrus (IFG) in the contralateral hemisphere, and the SMG in the ipsilateral hemisphere (Figure 3 and Table 1). Decoding accuracies obtained from each identified cluster were as follows: 73.8 ± 6.7% for the contralateral S2, 64.4 ± 4.7% for the contralateral IFG, and 61.8 ± 6.6% for the ipsilateral SMG. Interestingly, the bilateral SMG regions were identified in common across the two searchlight analyses.

**Table 1:** Searchlight analyses: identified brain regions in which the local activity patterns allowed to discriminate the three levels of stickiness intensity (*p* < 0.001 uncorrected, size >100).

Regions	Side	MNI coordinates			Cluster size	*T*	*Z*
		*x*	*y*	*z*			
Skin contact							
Supramarginal gyrus	Right	60	−28	42	138	5.94	4.31
-	Right	54	−28	52		4.71	3.72
Supramarginal gyrus	Left	−56	−28	32	270	5.50	4.11
*Postcentral gyrus*	Left	−60	−20	36		5.39	4.06
Supplementary motor area	Right	8	10	46	366	5.41	4.07
-	Left	−8	8	50		5.29	4.01
Glove contact							
Postcentral gyrus	Left	−52	−18	30	739	7.20	4.81
*Supramarginal gyrus*	Left	−56	−28	32		6.00	4.34
Inferior frontal gyrus	Left	−50	16	24	217	6.39	4.50
*Middle frontal gyrus*	Left	−34	36	40		5.05	3.89
Supramarginal gyrus	Right	54	−28	52	179	5.85	4.27
-	Right	60	−28	42		5.71	4.21
Cross-decoding							
Inferior frontal gyrus	Left	−52	16	24	250	6.63	4.60
-	Left	−54	24	14		5.64	4.18
Angular gyrus	Right	46	−60	34	144	6.00	4.34
-	Right	54	−62	30		5.84	4.27
Angular gyrus	Left	−34	−74	42	162	5.89	4.29
-	Left	−30	−70	50		5.56	4.14

*Cluster size indicates N voxels, T indicates peak t-values, Z indicates peak z-values. Entries without brain region name labels or in italics indicate sub-peak within the cluster named above*.

### Searchlight Analyses Between Touch Conditions

**Figure 4:**
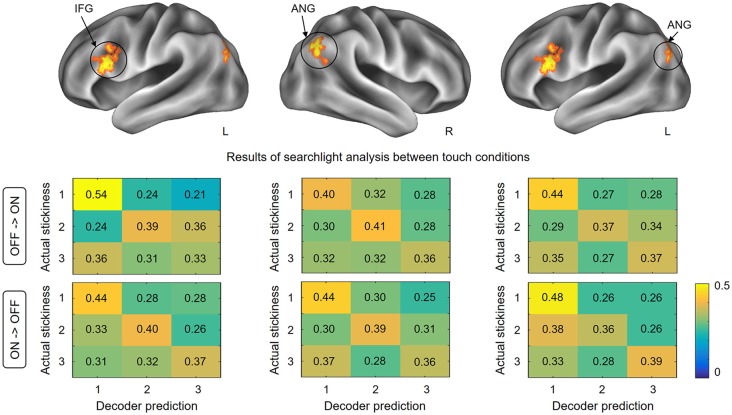
Results of searchlight analysis between touch conditions. The searchlight MVPAs identified voxel clusters showing significant decoding performances for the stickiness intensity classification independent of the touch condition. The cross-condition decoding analysis identified the IFG in the contralateral hemisphere and the bilateral angular gyri, in which neural activation patterns allowed stickiness intensity discrimination regardless of the touch conditions. The lower part of the figure shows the confusion matrices for each direction of classification analyses in identified clusters. Abbreviations: ANG, angular gyrus; IFG, inferior frontal gyrus.

This searchlight analysis aimed to determine the neural representations of stickiness intensity independent of the touch condition. Using a cross-condition classification method, we further investigated whether the local spatial patterns of activation allow the successful discrimination of intensity levels regardless of the touch condition mediating the stickiness. This searchlight analysis revealed three significant clusters (*p* < 0.001 uncorrected, size >100; Figure 4 and Table 1): these clusters were located in the IFG in the contralateral hemisphere and the bilateral angular gyri. Decoding accuracies obtained from each identified cluster were as follows: 41.2 ± 3.7% for the contralateral IFG, 39.3 ± 4.4% for the ipsilateral angular gyrus, and 40.2 ± 3.6% for the contralateral angular gyrus. These decoding accuracies were still significantly higher than the chance level (33.3%), but the relative performances were worse than the within touch condition cases.

To test whether the classification patterns were indeed similar across the two touch conditions in our decoding analysis, we computed confusion matrices for each cross-validation step. Figure 4 shows the group-level confusion matrices for each identified cluster. Rows of the confusion matrix indicate the true intensity label and columns indicate predictions of the classifier. Diagonal cells thus represent correct predictions and off-diagonal cells indicate misclassifications. Using two-sample *t*-tests with z-scored accuracies, we statistically compared the values of the confusion matrices of two folds directly against each other. The results revealed that no region performed well in one fold and poorly in the other fold (all *p*s > 0.26). This confirms that the classification patterns for each fold of decoding were similar and that there was no important information lost during the averaging process of cross-validation steps.

It is also noticeable that the highest correct prediction cells for each matrix were consistently observed for the stickiness level 1 (with one exception, i.e., for the confusion matrix in the case of decoding from glove off to glove on condition in the ipsilateral angular gyrus). A one-way ANOVA on the classification performance revealed that there was a significant effect of stickiness intensity levels (*F*_(2,159)_ = 8.38, *p* < 0.01). A Tukey *post hoc* test showed that the correct prediction rates were significantly higher when a stimulus of stickiness level 1 was provided, compared to when stickiness levels 2 and 3 were provided (all *p*s < 0.02). There was no significant difference between the stickiness levels 2 and 3 (*p* = 0.39).

## Discussion

In the current study, we investigated how the human brain represents the stickiness of a surface when this surface (an adhesive tape) was either touched directly with a fingertip or indirectly with a glove. Results of the subjective rating during the scanning sessions indicated that all participants could discriminate between the different stickiness levels of the stimuli in both touch conditions and perceived levels 2 and 3 as less sticky in the glove contact than the skin contact condition. The searchlight analyses revealed that there were brain regions that were contributing to the intensity discrimination in both touch conditions. These regions included the bilateral SMG (extending to the S2), the SMA, and the contralateral IFG. In the cross-decoding searchlight analysis, we observed that neural activity patterns in the contralateral IFG and the bilateral angular gyri contained similar stickiness intensity information irrespective of how the sticky sensations were evoked. Furthermore, we examined the classification patterns for each cross-validation fold using confusion matrices. Irrespective of the decoding direction (i.e., classifier decoding from skin contact to glove contact condition, and vice versa) and the brain regions, the classification performance of level 1 was consistently higher than those of level 2 and 3. This result indicates that the intensity level 1 is encoded in more similar neural activity patterns than the level 2 and 3 regardless of how participants touched the sticky stimuli. Interestingly, in line with this observation, the behavioral responses during the scanning sessions showed that participants perceived the stickiness level 1 at a similar intensity regardless of touch conditions, while they perceived levels 2 and 3 differently depending on the touch condition. Our results, therefore, suggest that the similarity of perceived stickiness intensity across both conditions is closely related to the neural encoding patterns, supporting the hypothesis that the brain encodes perceived stickiness intensity.

One of the main findings in this study is that neural activities in the SMG and the S2 were consistently involved in tactile intensity encoding when participants touched sticky surfaces not only with the bare skin of a fingertip but also with a gloved hand. Many human neuroimaging studies have shown that both SMG and the S2 are central for somatosensory information processing such as tactile perception and discrimination (Bodegård et al., 2001; Lamp et al., 2019). These two regions are located close to each other in the parietal operculum. Previous studies have reported the co-activations of these regions in a wide range of experimental paradigms (Burton et al., 2008; Jung et al., 2009) and demonstrated the functional connectivity of these areas (Eickhoff et al., 2006). According to these previous findings, the co-identification of SMG and S2 from our experimental paradigm is highly likely due to the similar functional role in somatosensory information processing. In addition to the regions in the parietal lobe, our results suggest that (1) the SMA and (2) the IFG are involved in tactile stickiness perception. (1) This is suggested by our searchlight analysis for the skin contact condition which found distinct response patterns to the different tactile intensities in the SMA. Previously the SMA has been well known as a region responsible for the control and the planning of movements (Matsuzaka and Tanji, 1996) and several studies have reported SMA activations during active tactile exploration tasks (Simões-Franklin et al., 2011; Kim et al., 2015a). Therefore, we cannot exclude the possibility that the neural activations that we observed in the SMA might at least partly be influenced by the participants’ finger movements actively detaching their finger from the sticky surfaces and by the accompanying muscle and joint afferents. In line with this speculation, a previous human fMRI study found significant neural activities in the SMA during a roughness categorization task, but the authors considered this activity to reflect the motor components of the active task rather than tactile roughness categorization *per se* (Simões-Franklin et al., 2011). Further, technically more difficult studies using passive touch instead of active touch would help clarify this matter. (2) The contralateral activation of the IFG was identified in the searchlight analysis for the glove contact condition and the cross-decoding searchlight analysis, suggesting that this area also encodes stickiness intensity information. While the IFG is widely known to be involved in speech and language processing, several studies have suggested, following our findings, that this region is also associated with somatosensory processing (Hagen et al., 2002; Zhang et al., 2005). For example, a human PET study explored the involvement of ventral frontal regions in the somatosensory processing and revealed that somatosensory stimulation elicited neural activities in the posterior IFG and adjacent anterior frontal operculum (Hagen et al., 2002). Moreover, Kostopoulos and colleagues observed significantly increased activities in those areas when the disambiguation of tactile information in memory was required (Kostopoulos et al., 2007). Given our findings that both the somatosensory cortices and the IFG are involved in tactile stickiness perception, it is interesting to note that these authors demonstrated that the mid-ventrolateral prefrontal region, which includes the IFG, interacts functionally with the S2. Intriguingly, all identified brain regions in our study are known as substantial parts of the tactile working memory network (Preuschhof et al., 2006; Spitzer et al., 2010). In our fMRI experiment, after detaching the finger from the surface, the participants needed to maintain its stickiness information in memory so that this information can be processed and rated before the participants were able to provide their responses. Thus, the obtained brain signals in our study might be attributed to the encoded tactile working memory contents.

Figure 4 indicates that the identified brain regions (bilateral angular gyri and the contralateral IFG) encode the stickiness level 1 in a more similar pattern of neural activity in both direct and indirect touch conditions than it is the case for levels 2 and 3. This observation can be explained by the participants’ stickiness ratings (Figure 2). Participants perceived intensity level 1 similarly with or without a glove, but intensity levels 2 and 3 were perceived differently. When removing the gloved finger from a sticky surface, there would be some changes in the contact between the finger skin and the glove, which can also signal stickiness information. Based on the observation that participants perceived intensity levels 2 and 3 as less sticky when they were touched with a gloved hand than by a bare hand, the tactile intensity perception may be related to the interaction between stickiness and the gloved or bare hand. This difference in ratings together with the classification patterns of cross-decoding analyses supports the hypothesis that the neuronal populations within identified regions encode the perceived stickiness intensity level, *not* the physical intensity level.

It is noteworthy that we found no significant activation in S1. This observation is unexpected because previous neuroimaging studies have consistently shown that S1 processes tactile intensity coding (Bourgeon et al., 2016; Case et al., 2016). One possible explanation is that our searchlight MVPA looked for brain regions contributing to tactile intensity discrimination, not tactile intensity perception. Contrary to intensity perception, intensity discrimination is focused on the difference between two distinct sensations. Consistent with this idea, the aforementioned study by Case and colleagues reported an inverse correlation between changes in tactile discriminability and intensity ratings for S1 (Case et al., 2016). Another possibility is individual variation in S1. A previous fMRI study attempted to discriminate the location of the vibrotactile stimulation and observed no significant activation in S1, but found significant activation in adjacent cortical regions (Kim et al., 2015b). They examined individual variations in S1 responses and hypothesized that individual variations in S1 may be too large to decode stimulated locations with sufficient significance. Therefore, the complexity of individual S1 representations can make it difficult to identify patterns in S1, as well as that an individual’s blood vessel anatomy can obscure fMRI signals.

Our study has several limitations. First, the relatively small number of participants (*n* = 18) and unbalanced gender ratio (14:4) reduce the generalizability of the present findings. It could be beneficial to include gender in the statistical model. However, since this study has extremely unbalanced gender groups, adding gender as a parameter might have deteriorated statistical precision and unnecessarily decreased the power of the analysis. For these reasons, we decided not to consider gender in the fMRI analysis. Moreover, the potential influence of posture differences in tactile explorations cannot be ruled out. Contrary to the training set, during the image acquisition, participants explored the surface texture in the supine position on the scan table. Thus, applying the same amount of pressure could be difficult due to posture difference. Lastly, we could not fully control the potential confounds of MVPA. According to the concerns raised by Todd and colleagues (Todd et al., 2013), several variables of no interest (e.g., reaction time and individual differences) have a significant influence on the MVPA results.

The present study investigated neural representations of tactile stickiness information in response to the exploration of surface texture. To the best of our knowledge, this is the first attempt to investigate brain activation patterns evoked from two distinct touch conditions, for example, touch with bare and gloved hands. This topic should be more extensively investigated to extend our knowledge regarding the direct and indirect ways of surface texture perception (LaMotte, 2000; Klatzky et al., 2003). A study about multisensory aspects of the surface stickiness in the human brain should be conducted in the future.

## Data Availability Statement

Both the fMRI images and codes related to this publication will be shared upon request with adequate reasons such as to validate the reported findings and to perform a new analysis (Junsuk Kim, junsuk.kim@deu.ac.kr).

## Ethics Statement

The studies involving human participants were reviewed and approved by University Clinics Tübingen (649/2016BO2). The patients/participants provided their written informed consent to participate in this study.

## Author Contributions

JK and IB conceived the project and reviewed and edited the manuscript. JK analyzed results and wrote the original draft of the manuscript. HB supervised the project.

## Conflict of Interest

The authors declare that the research was conducted in the absence of any commercial or financial relationships that could be construed as a potential conflict of interest.
